# Mechanisms of partial hydrogen sorption reversibility in a 3NaBH_4_/ScF_3_ composite

**DOI:** 10.1039/c8ra00429c

**Published:** 2018-03-01

**Authors:** Ning Zhao, Jianxin Zou, Xiaoqin Zeng, Wenjiang Ding

**Affiliations:** National Engineering Research Center of Light Alloy Net Forming, State Key Laboratory of Metal Matrix Composites, Shanghai Jiao Tong University Shanghai 200240 P. R. China zoujx@sjtu.edu.cn +86-21-34203730 +86-21-54742381; Shanghai Engineering Research Center of Mg Materials and Applications, School of Materials Science and Engineering, Shanghai Jiao Tong University Shanghai 200240 P. R. China

## Abstract

A new hydrogen storage composite containing NaBH_4_ and a 3d transition metal fluoride, 3NaBH_4_/ScF_3_, was synthesized *via* ball milling. The composite shows no reaction during milling and its dehydriding process can be divided into three steps upon heating: (i) partial substitution of H^−^ by F^−^ in NaBH_4_ to form NaBH_*x*_F_4−*x*_ at the early stage, releasing about 0.19 wt% of hydrogen; (ii) formations of Na_3_ScF_6_, NaBF_4_ and ScB_2_ through the reaction between NaBH_4_ and ScF_3_, with 2.52 wt% of hydrogen release and a dehydriding activation energy of 162.67 kJ mol^−1^ H_2_; (iii) further reaction of residual NaBH_4_ and Na_3_ScF_6_ to form NaF, B and ScB_2_, with a dehydriding activation energy of 169.37 kJ mol^−1^ H_2_. The total hydrogen release of the composite reaches 5.54 wt% at 530 °C. The complete dehydrided composite cannot be rehydrogenated while the products after the second dehydriding step can be hydrogenated with an absorption activation energy of 44.58 kJ mol^−1^ H_2_. These results demonstrate that by adding 3d transition metal fluorides into NaBH_4_, a partial reversibility in NaBH_4_ can be achieved.

## Abstract 2

The dehydrogenation of 3NaBH_4_/ScF_3_ involves three consecutive processes, and the second dehydrogenation step has reversibility.

## Graphical abstract

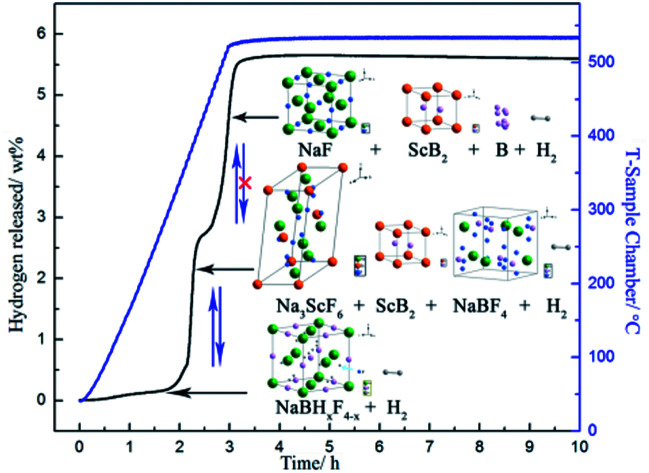

## 1 Introduction

Hydrogen is one of the most promising alternative and attractive clean energy sources that can substitute fossil fuels, with sufficient energy density and environment-friendliness.^1,2^ Nevertheless, almost a century since the concept of “hydrogen economy” was introduced by Jules Velne,^3,4^ it is still challenging to find reliable, flexible and cost-efficient hydrogen media for on-board, stationary and portable applications.^5,6^ In the past few decades, great attention has been paid to both hydrogen production technologies and a variety of hydrogen storage methods,^7–10^ including the use of different compounds,^11,12^ especially complex hydrides, of which borohydrides are typical representative.^13–15^ These solid-state hydrogen storage materials offer some advantages over high pressure gaseous storage and low temperature liquid storage, such as high capacity, high safety, and low cost. In particular, alkali metal borohydrides are regarded as possible hydrogen carries, ascribed to their contribute to their high gravimetric and volumetric hydrogen density, together with good stability.^16^ However, current research results showed that few technologies regarding the use of metal borohydrides as hydrogen storage carriers are able to fulfill the requirements established by the US Department of Energy.^17^

Early investigations into borohydrides have shown that, compared to other borohydrides such as LiBH_4_ and KBH_4_, NaBH_4_ is stable under alkaline conditions, and undergoes hydrolysis through the following reaction:^18^

1 NaBH_4_ + 4H_2_O → NaBO_2_·2H_2_O + 4H_2_

However, the use of NaBH_4_ as a hydrogen generator through hydrolysis faces several issues related to the catalyst durability, and/or poisoning, as well as storage vessels.^19–21^ In contrast, thermal decomposition of NaBH_4_ has emerged as potential alternative method for hydrogen storage. J. Urgnani *et al.* investigated the thermal decomposition behaviors of NaBH_4_, and proposed that NaBH_4_ would decompose in two steps according to the following reactions:^22^2
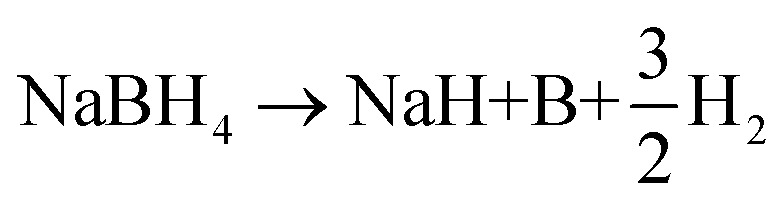

3 NaBH_4_ → Na + B + 2H_2_

For the sake of improving the thermodynamic and kinetic properties of the thermal decomposition of NaBH_4_, many strategies have been taken, such as adding catalyst, building thermodynamic destabilization system, nano-engineering and chemical modifications.^13,23–25^ For instance, NaBH_4_/MgH_2_ system could fulfil its decomposition before melting due to the formation of MgB_2_ in the system.^26^ Czujiko *et al.* suggested that pure Mg could lower down the desorption temperature of NaBH_4_ through catalytic effect.^27^ In addition, the decomposition of NaBH_4_ may occur at lower temperatures with some reversibility through the addition of Ni-based catalysts,^28^ which facilitates hydrogen release and improves the reversibility to some extent.^29^ Moreover, the chemical reaction that regenerates borohydrides from metal–borides occurs much easier over the regeneration from boron since less energy is required for breaking the chemical bond between B–M (M means metal) relative to the B–B's.^30^

In previous works, many research works regarding hydrogen storage composite systems have been carried out, and some of them have explored how rare earth element (RE) addition can effect the thermodynamics and kinetics properties of metal borohydrides based systems, *i.e.*, NaBH_4_–YF_3_,^31^ NaBH_4_–ScCl_3_,^32^ LiBH_4_–YCl_3_,^33^*etc.* In particular, hydrogen sorption reversibility was achieved in 3NaBH_4_/LnF_3_ systems with good thermodynamic and kinetic properties.^34^

Considering that scandium lies in the III B column of the periodic table as the lanthanide elements, we attempt to prepare a new hydrogen storage system, 3NaBH_4_/ScF_3_, through ball milling method. Our previous investigations on NaBH_4_–MF_3_ (M = metal) systems proved that the molar ratio of 3 : 1 was the best one, *i.e.* 3NaBH_4_/LnF_3_ (Ln = La, Ce, Nd, Gd, Yb),^34^ 3NaBH_4_/YF_3_,^31^ 3NaBH_4_/PrF_3_ and 3NaBH_4_/HoF_3_.^35,36^ If the molar ratio of NaBH_4_ to MF_3_ is higher than 3 : 1, some NaBH_4_ will be left after the desorption due to the incomplete reaction. While if the ratio is less than 3 : 1, MF_3_ is excessive, and the overall hydrogen sorption capacity is reduced since MF_3_ can neither release nor absorb hydrogen. Consequently, the ratio of NaBH_4_ to ScF_3_ is set as 3 : 1 in the present work. We conducted a detailed study of hydrogen sorption behaviors of the 3NaBH_4_/ScF_3_ system, and proposed mechanisms of hydrogen sorption in this composite, depending on experimental analyses.

## 2 Experimental

### 2.1 Sample preparation

NaBH_4_ (Aladdin Reagent Database Inc., 96%) and ScF_3_ (Aladdin Reagent Database Inc., 98%) were used as starting materials without further purification and mixed in the molar ratio of 3 : 1 in a planetary ball miller whose type is QM-1SP2. The stainless steel vessel with 100 ml volume was used to load 0.3928 g of NaBH_4_ and 0.3530 g of ScF_3_ powders together with 25 stainless steel balls (diameter of 5 mm, average weight of 0.8950 g each). The ball to powder weight ratio is approximately 30 : 1. Ball milling is conducted at a rotation speed of 400 rpm for 180 min. The prearrangement, manipulation and storage of specimen were carried out in an Ar-filled Lab 2000 glove box (Etelux inert gas system Co., Ltd.) in which neither moisture nor oxygen concentration beyonds 10 ppm.

### 2.2 Characterization

The analyses of phase composition for the ball milled, dehydrogenated and rehydrogenated samples were accomplished using an X-ray diffraction apparatus (D/max 2550VL/PC), equipped with a Cu-Kα radiation source. For XRD tests, composite powder at different states were kept into specific sample holders with arched glass on both sides and airtight PVC tape on the top, to isolate them from air. Meanwhile, the broad peak at around 2*θ* = 15° in the patterns is caused by the tape. Using a Spectrum Nicolet iS5 produced by Thermo Fisher Scientific Inc., Fourier transform infrared spectroscopy (FTIR) tests were performed on samples with different states in an Ar filled glove box. In principle of volumetric methods, we carried out temperature-programmed-desorption (TPD) measurements on 0.2 g of the 3NaBH_4_/ScF_3_ composite sample from room temperature to about 530 °C under an initial vacuum condition, with a heating rate *R*_H_ of 3 °C min^−1^. Evaluations of hydrogen absorption performance were implemented for 10 h at various temperatures under around 3.2 MPa H_2_ pressure.

Dehydrogenation behaviors of composites were determined by synchronous thermal analyzer (Differential Scanning Calorimetry/Thermal Gravimetry, DSC/TG), in a Netzsch, STA 449 F3 Jupiter equipment, in which *R*_H_ = 3 °C min^−1^, 5 °C min^−1^, 7 °C min^−1^ and 10 °C min^−1^, respectively, starting from room temperature to about 500 °C, under the protection of 0.1 MPa Ar flow. To compare the properties of hydrogen storage performances of different composites, data of weight percent considering hydrogen release and uptake during the tests was assessed based on the samples' initial weight value.

## 3 Results and discussions

### 3.1 Dehydrogenation of the 3NaBH_4_/ScF_3_ composite

**Fig. 1:**
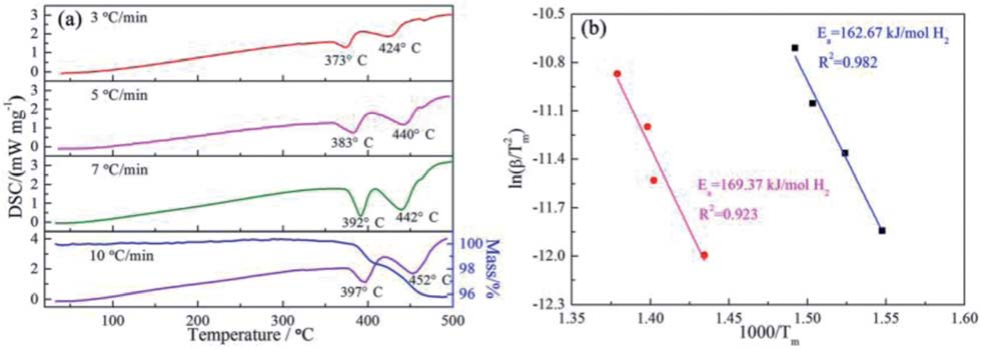
DSC curves of 3NaBH_4_/ScF_3_ composite samples under heating rates of 3 °C min^−1^, 5 °C min^−1^, 7 °C min^−1^ and 10 °C min^−1^ and TG profile at the heating rate of 10 °C min^−1^ (a) and the corresponding Kissinger plots for the two major endothermic desorption steps (b).

The effect of the ScF_3_ addition on the hydrogen desorption behaviors of NaBH_4_ was examined by DSC measurements at different *R*_H_, namely 3 °C min^−1^, 5 °C min^−1^, 7 °C min^−1^ and 10 °C min^−1^, as well as TG measurement at the *R*_H_ of 10 °C min^−1^, as Fig. 1 showed.

Two major endothermic peaks upon heating appeared on the DSC curves, indicating that two main desorption steps took place during dehydrogenation. According to the starting point of dehydriding in DSC profiles, the first major dehydrogenation begins at 356 °C with *R*_H_ = 3 °C min^−1^ heating rate condition. In contrast, pure NaBH_4_ shows a dehydrogenation temperature of 517 °C under the same condition.^31^ In addition, a subsequent broad endothermic peak was also recorded. TG curve obtained at the heating rate of 10 °C min^−1^ shows that the total mass loss reaches 4.10 wt% at 500 °C. According to the DSC/TG profiles, the dehydrogenation enthalpies of the first and second major desorption reactions are determined to be 27.43 ± 5 kJ mol^−1^ H_2_ and 29.54 ± 2 kJ mol^−1^ H_2_, respectively.

The apparent dehydrogenation activation energy (*E*_a_) of the 3NaBH_4_/ScF_3_ sample could be determined using the Kissinger method,^37^ as described below:4
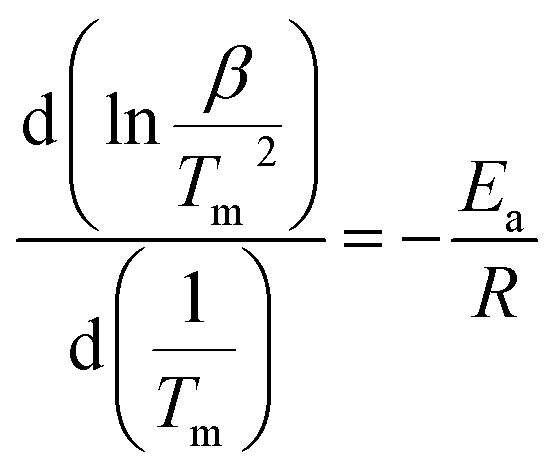
where heating rate (*β*), peak temperature (*T*_m_), and gas constant (*R*) show a specific relationship. Table 1 gives the peak temperatures in DSC curves at various *R*_H_ obtained from Fig. 1a. The fitting plot displays that ln(*β*/*T*_m_^2^) and 1/*T*_m_ have good linearity, as shown in Fig. 1b. According to eqn (4), the *E*_a_ value is calculated to be 162.67 kJ mol^−1^ H_2_ and 169.37 kJ mol^−1^ H_2_ for the first major desorption step and second major desorption step, respectively.

**Table 1:** The DSC peak temperatures of the 3NaBH_4_/ScF_3_ at different heating rates under 0.1 MPa argon atmosphere

Sample	Heating rate/°C min^−1^	Temperature of peaks/°C	
3NaBH_4_/ScF_3_	3	373	424
	5	383	440
	7	392	442
	10	397	452

**Fig. 2:**
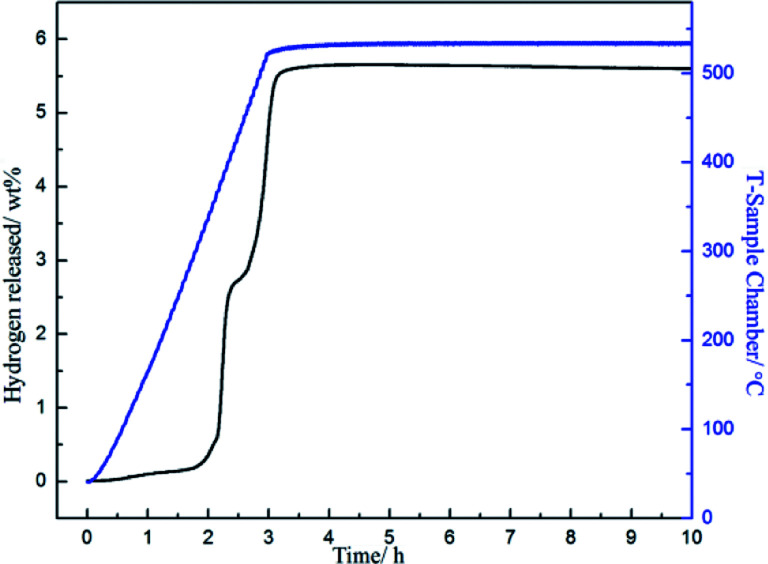
TPD profile of the 3NaBH_4_/ScF_3_ sample at a heating rate of 3 °C min^−1^.

To obtain further information of dehydrogenation process of the target system, TPD measurement was performed on the ball-milled composite with a constant *R*_H_ of 3 °C min^−1^ from ambient temperature to 530 °C and the results are shown in Fig. 2. The results exhibit that 3NaBH_4_/ScF_3_ composite has an appropriate onset dehydrogenation temperature, which is actually lower than 200 °C in vacuum. Meanwhile, the figure shows that the desorption behavior may be subdivided into three consecutive processes, with a small amount of hydrogen (∼0.19 wt%) released at temperature lower than 310 °C in the initial process, and the latter two steps range from 310 °C to 420 °C, and above 420 °C, releasing about 2.52 wt% and 2.83 wt% of hydrogen, respectively. In comparison to the DSC measurements, the second and third desorption steps shown in TPD profile should correspond to the two major endothermic peaks on DSC curves.

The entire hydrogen release obtained from the experimental process up to 530 °C is 5.54 wt%, which is over 95% of the theoretical hydrogen content. Previous study shows that during dehydrogenation process of pure NaBH_4_ under the same condition, only 0.68 wt% weight loss is observed when heated up to 482 °C.^19^ Thus, the hydrogen desorption properties of NaBH_4_ were significantly promoted by the addition of ScF_3_. However, from Fig. 1, there is no endothermic peak present from ambient to 300 °C in DSC curves. C. Bonatto Minella reported a related phenomenon and the difference between DSC analyses and volumetric measurements was ascribed to dissimilar experimental conditions.^38^

**Fig. 3:**
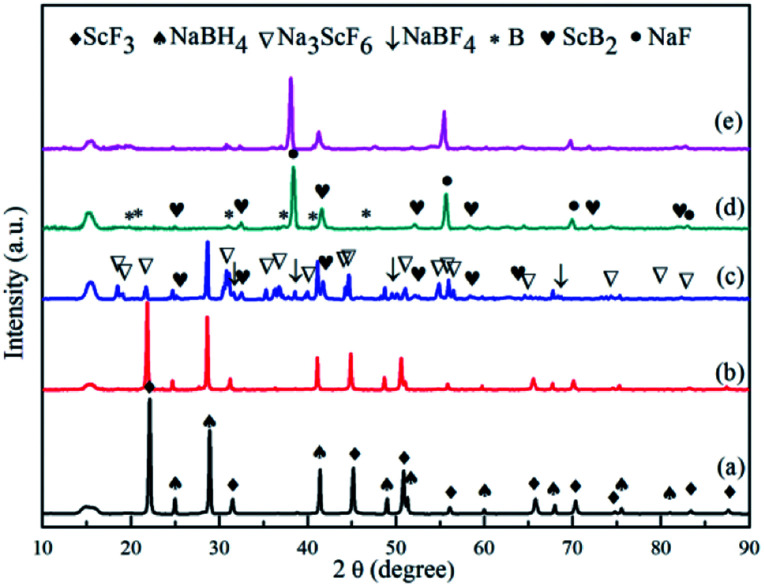
XRD patterns of the 3NaBH_4_/ScF_3_ composite after ball milling (a), dehydrogenated at 300 °C (b), dehydrogenated at 420 °C (c), fully dehydrogenated at 530 °C (d) and rehydrogenated at 420 °C (e).

**Fig. 4:**
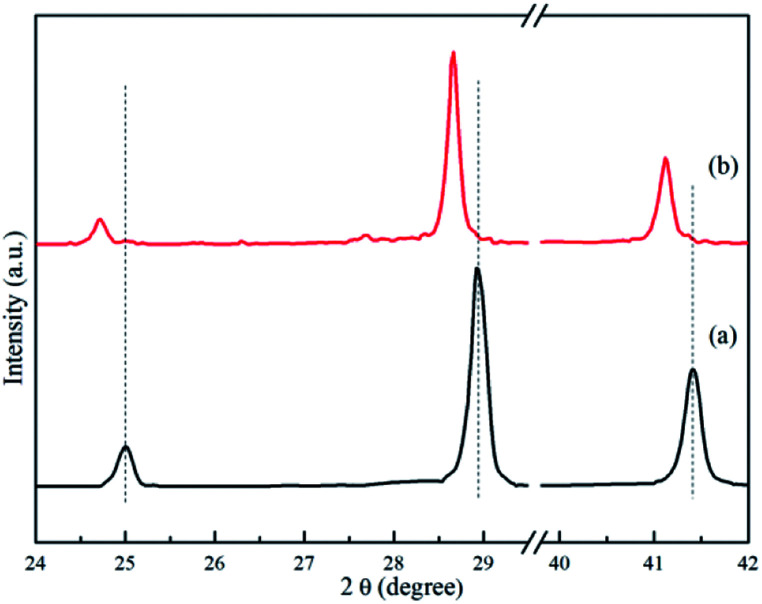
XRD patterns of 3NaBH_4_/ScF_3_ composite after ball milling (a) and after dehydrogenation at 300 °C (b) in the range of low diffraction angle.

XRD analyses were performed on samples treated under a series of controlled conditions in order to have a better understanding of mechanisms of de/rehydrogenation in the 3NaBH_4_/ScF_3_ composite, as shown in Fig. 3. The results show that no new product can be found in the sample after ball milling, except NaBH_4_ (JCPDS no. 09-0386) and ScF_3_ (JCPDS no. 46-1243), which means that only physical mixing takes place during milling process. Before dehydrogenation at various temperatures of 300 °C, 420 °C and 530 °C, the loaded sample container was first placed under a 4.5 MPa H_2_ pressure, then followed by a quick temperature rising and heat preservation. At last, the sample chamber was evacuated and powders were treated at the expected temperature for 3 h. After dehydrogenation at 300 °C under vacuum, it can be clearly seen in Fig. 4 that diffraction peaks from NaBH_4_ in the 3NaBH_4_/ScF_3_ composite shifted slightly from high angle side to lower angle side, demonstrating a lattice expansion of NaBH_4_ after the first dehydrogenation.

On the basis of the XRD patterns and using the RIR analysis, the lattice parameters of NaBH_4_ in ball milled sample is calculated to be: *a* = 0.6165 nm, *b* = 0.6184 nm, *c* = 0.6153 nm and *α* = *β* = *γ* = 90°. After the first step dehydrogenation at 300 °C, the lattice constants of NaBH_4_ changed into *a* = 0.6185 nm, *b* = 0.6225 nm, *c* = 0.6166 nm and *α* = *β* = *γ* = 90°. Such a lattice expansion of NaBH_4_ might be attributed to the fact that H^−^ was partially substituted by F^−^ in the unit cell. Similar phenomenon was also observed in some previous works,^29,39–43^ for which the lattice expansion was attributed to the formation of an intermediate compound NaBH_*x*_F_4−*x*_. In the present work, the formation of NaBH_*x*_F_4−*x*_ occurred at the first desorption step between NaBH_4_ and ScF_3_, together with releasing small amount of hydrogen, as also seen in the 3NaBH_4_/NdF_3_, 3NaBH_4_/PrF_3_, 3NaBH_4_/HoF_3_ systems,^19,35,36^ and was regarded as an energy favorable process in theory.^44^ Upon heating at temperatures higher than 310 °C, along with the reduction of NaBH_4_ and disappearance of ScF_3_ (Fig. 3c), Na_3_ScF_6_ appeared in the system, indicating that a major reaction between NaBH_4_ and ScF_3_ occurred. It has been also indicated by Radovan Cerny *et al.* that in the case of NaBH_4_/ScCl_3_ system, Na_3_ScCl_6_ and NaSc(BH_4_)_4_ formed as a result of the reaction between NaBH_4_ and ScCl_3_.^32^ Chong *et al.* reported that the reaction occurred at 250 °C between NaBH_4_ and HoF_3_ could produce NaHo(BH_4_)_4_ and NaHo_2_F_7_ phases.^36^ In the 3NaBH_4_/ScF_3_ composite, NaSc(BH_4_)_4_ might form upon heating and then decomposed to ScB_2_ and H_2_. Based on the XRD analysis, the second dehydrogenation step before 420 °C can be described as:

5 27NaBH_4_ + 20ScF_3_ = 8Na_3_ScF_6_ + 12ScB_2_ + 54H_2_ + 3NaBF_4_

Such a reaction has a theoretical hydrogen release of 2.53 wt%, close to the value measured from TPD for the second step dehydrogenation. At around 500 °C, the remaining NaBH_4_ reacts with Na_3_ScF_6_ and NaBF_4_ to produce NaF, ScB_2_ and B, as shown in the indexed XRD pattern of Fig. 3d. Thus, the third dehydrogenation step can be described as follows:

6 33NaBH_4_ + 8Na_3_ScF_6_ + 3NaBF_4_ = 60NaF + 8ScB_2_ + 66H_2_ + 20B

with a theoretical hydrogen desorption value of 3.08 wt%. This value is also close to what is observed in the TPD analysis for the third dehydrogenation step. According to Garroni *et al.*,^45^ Na_2_[B_12_H_12_] is usually a byproduct during desorption of NaBH_4_ based composites, which forms in an intermediate step and is still present at the end of reaction. Na_2_[B_12_H_12_] is found to be a stable byproduct and cannot be re-hydrogenated to NaBH_4_, thus is regarded as an unfavorable product for the reversibility.^46,47^ However, the Na_2_[B_12_H_12_] phase was not found in the XRD pattern of the partial or the complete dehydrogenated 3NaBH_4_/ScF_3_ samples, which means that very small amount or even no such a byproduct was generated during the decomposition of the 3NaBH_4_/ScF_3_ composite.

### 3.2 Rehydrogenation in the 3NaBH_4_/ScF_3_ composite

Fig. 3e shows the XRD pattern of the complete dehydrogenated 3NaBH_4_/ScF_3_ composite (530 °C for 3 h) that is maintained under a pressure of 3.2 MPa H_2_ at 420 °C for 10 h. The pattern shows no change compared to that of the complete dehydrogenated composite, which means that the latter one has no hydrogen absorption ability.

**Fig. 5:**
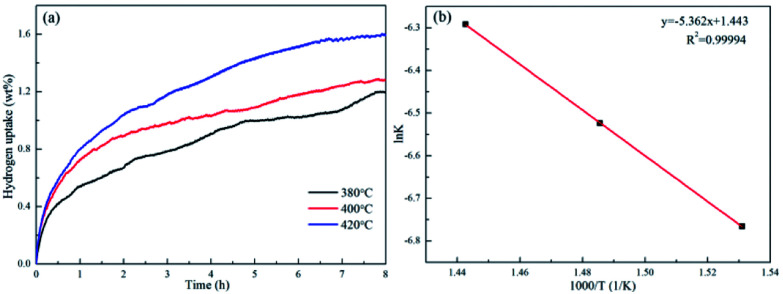
Isothermal hydrogen absorption curves for the partially dehydrogenated 3NaBH_4_/ScF_3_ composite sample at temperatures of 380 °C, 400 °C and 420 °C (a) and the ln *k*-1000/*T* fitting plot (b).

As the complete dehydrogenated 3NaBH_4_/ScF_3_ composite shows no reversibility, the hydrogen absorption after the second dehydrogenation step was attempted to study the possible reversibility. The profiles of hydrogen absorption are given in Fig. 5a, which are obtained under the condition of 380 °C, 400 °C and 420 °C at 3.2 MPa H_2_ pressure for the sample that has gone through dehydrogenation at 420 °C for 3 h. These curves clearly show the reversible hydrogen absorption of the partial dehydrogenated 3NaBH_4_/ScF_3_ composite: under the pressure of 3.2 MPa H_2_, a hydrogenation capacity of 1.59 wt% can be achieved at 420 °C for 8 h, while it can absorb 1.28 wt% at 400 °C and 1.19 wt% at 380 °C, respectively. By contrast, under 3.5 MPa hydrogen pressure, pure NaBH_4_ shows no hydrogen absorption at 400 °C.^36^

The hydrogenation activation energy (*E*_ab_) is generally utilized to discriminate kinetics of absorption, by analyzing the entire energy barriers of hydrogen absorption process. Based on the Johanson–Mehl–Avrami (JMA) model, the following equation can be used to evaluate absorption kinetics:^48^

7 ln[−ln(1 − *α*_A_)] = *η* ln *k* + *η* ln *t*

where *α*(*t*) is a function of time *t*, *k* is a parameter describing kinetic, *η* is the Avrami exponent which matches transformation mechanism. Then, the following Arrhenius equation is used to calculate *E*_ab_:8
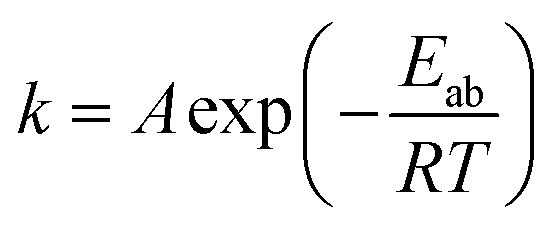
where *A* represents temperature-independent constant, *R* represents universal gas constant, and *T* represents the absolute temperature. The scheme of ln *k versus* 1000/*T*, which is shown in Fig. 5, displays a good linear relationship. Therefore, the *E*_ab_ value obtained from the slope is therefore estimated to be 44.58 kJ mol^−1^ H_2_ for the partially dehydrogenated 3NaBH_4_/ScF_3_ composite.

**Fig. 6:**
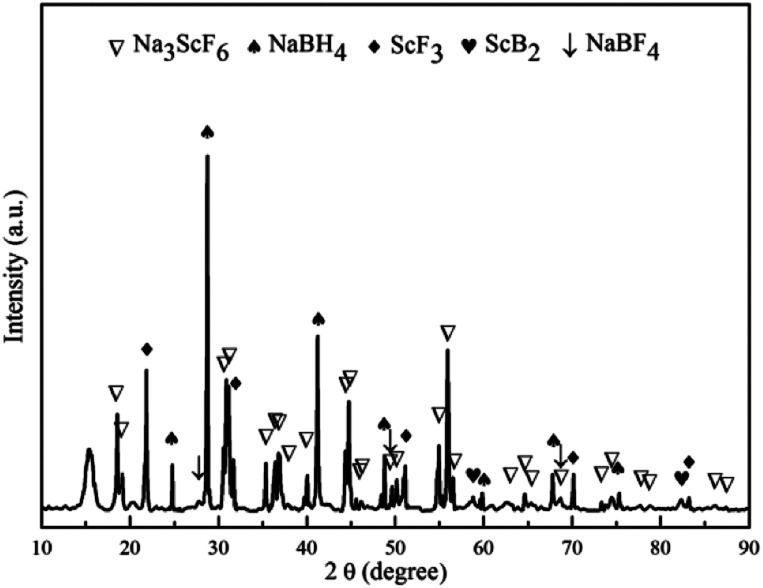
XRD pattern of the partially dehydrogenated 3NaBH_4_/ScF_3_ composite sample after rehydrogenation.

To elucidate the mechanism of hydrogen absorption in the partially dehydrogenated 3NaBH_4_/ScF_3_ composite, XRD analysis is carried out on the rehydrogenated sample and the result is shown in Fig. 6. The 3NaBH_4_/ScF_3_ sample was dehydrided at 420 °C for 3 h in vacuum and then rehydrogenated at 420 °C for 10 h under the pressure of 3.2 MPa H_2_. In Fig. 6, the diffraction peaks from NaBF_4_ and ScB_2_ phases became weaker and even disappeared along with the increment in peak intensities of NaBH_4_ and ScF_3_ as compared to those in Fig. 3c. Therefore, the hydrogen absorption in the partial dehydrogenated 3NaBH_4_/ScF_3_ composite follows exactly the reverse reaction path of the second step dehydrogenation. That is, the rehydrogenation consumes Na_3_ScF_6_, NaBF_4_ and ScB_2_, accompanied with the regeneration of NaBH_4_ and ScF_3_. Compared to the completely dehydrogenated 3NaBH_4_/ScF_3_ composite, the partially dehydrogenated composite contains Na_3_ScF_6_ and NaBF_4_ phases, indicating that these two phases play the key role for the rehydrogenation.

**Fig. 7:**
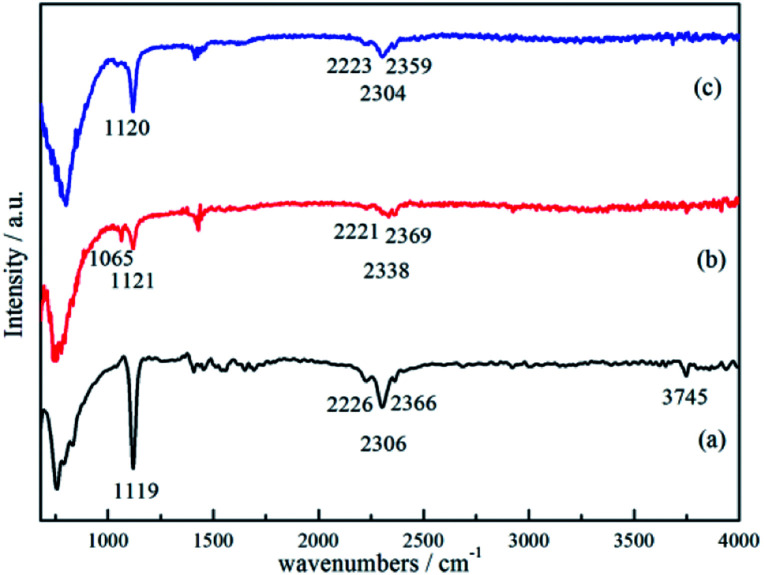
FTIR spectra of the 3NaBH_4_/ScF_3_ composite after ball-milling (a), half dehydrogenated 3NaBH_4_/ScF_3_ sample (b), rehydrogenation of partially dehydrogenated 3NaBH_4_/ScF_3_ sample (c).

The results of FTIR analyses for the ball-milled 3NaBH_4_/ScF_3_ sample, sample dehydrogenated at 420 °C for 3 h and corresponding products rehydrogenated at 400 °C for 3 h can be found in Fig. 7. In Fig. 7a, the FTIR spectrum of sample after ball milling has the signatures of B–H stretching band in the position of 2226 cm^−1^, 2306 cm^−1^ and 2366 cm^−1^, and B–Hbending band peak at 1119 cm^−1^, all of which are supposed to be originated from borohydride. These peaks are considered to be from NaBH_4_.^19^ However, it should be noted that, the height of those peaks, which represent the intensity of B–H bonds vibration from the [BH_4_]^−^ group, gradually become weaker as dehydrogenation reaction proceeds, indicating the decomposition of NaBH_4_, as seen at 1121 cm^−1^, 2221 cm^−1^, 2338 cm^−1^ and 2369 cm^−1^ in Fig. 7b. According to the work of D. Syamala,^44^ peak located at 1065 cm^−1^ can be marked as [BF_4_]^−^asymmetric stretching, indicating the formation of NaBF_4_ after the second step dehydrogenation, which is in good agreement with the XRD results (Fig. 3c). In Fig. 7c, the signatures of [BH_4_]^−^ bending at 1120 cm^−1^ and [BH_4_]^−^ stretching at 2223 cm^−1^, 2304 cm^−1^ and 2359 cm^−1^ were clearly revealed for the rehydrogenated sample and the intensity of these peaks increased, indicating the regeneration of NaBH_4_.^49^ Meantime, the peak from [BF_4_]^−^ asymmetric stretching disappeared after rehydrogenation, showing the consumption of NaBF_4_ along with rehydrogenation. Peaks at wave numbers between 1330 cm^−1^ and 1800 cm^−1^ (Fig. 7a) were subtracted from the unavoidable moisture absorption and atmospheric humidity absorbed by the sample during measurement, while peak located at around 3745 cm^−1^ was identified as stretching band vibration of O–H.^50^

### 3.3 Mechanisms of hydrogenation in 3NaBH_4_/ScF_3_ composite

It is shown in the present work that the hydrogen storage performance of NaBH_4_ can be effectively improved by introducing ScF_3_ as a reagent. In particular, rehydrogenation can be achieved in the partially dehydrogenated 3NaBH_4_/ScF_3_ composite. Analyses revealed that both Sc^3+^ cation and F^−^ anion show irreplaceable importance during the re/dehydrogenation processes of the composite. Firstly, F anion can replace H anion in the initial process of dehydrogenation, from NaBH_4_ to form NaBH_*x*_F_4−*x*_. Then, Sc cation loses electron to form ScB_2_, in which Sc cation has the calculated valence of +4.08,^51^ rather than served as a three-valent cation. This is accompanied with the formation of hydrogen gas. Meanwhile, during the second dehydrogenation step, a portion of F anions from ScF_3_ incorporate into Na_3_ScF_6_ crystallites, which might serve as the nucleation center for the growth of other products.

It has been established that the regeneration of NaBH_4_ in the NaBH_4_–MF_*x*_ systems is associated with electronegativity (*χ*_p_) of the metal cations.^52^ Previous works have shown that after adding transition metal fluorides into NaBH_4_ based composites, when the Pauling's electronegativity of the transition metal lies in around between 1.23–1.54, hydrogen sorption reversibility has larger thermodynamic tendency to occur.^52^ The *χ*_p_ value of Sc^3+^ is 1.415, which lies in such specific range, thus the regeneration of NaBH_4_ in the dehydrided 3NaBH_4_/ScF_3_ system is favorable.^53^

Using a database of density functional theory,^54–56^ the enthalpies of desorption reactions are calculated to be 41.01 kJ mol^−1^ H_2_ for the second dehydriding step, and 43.31 kJ mol^−1^ H_2_ for the final step. These values are comparably higher than the values obtained from DSC/TG analyses, but significant lower than that of pristine NaBH_4_ (108 ± 3 kJ mol^−1^ of H_2_).^57^ The differences between the calculated and measured enthalpies can be explained by fact that H^−^ was partially substituted by F^−^ in NaBH_4_, which is also observed in other NaBH_4_ based systems containing fluorides.^58^ However, the enthalpy for complete dehydrogenation is still fairly high, about 56.97 kJ mol^−1^ H_2_ calculated from DSC analyses. Consequently, the rehydrogenation of the complete dehydrided 3NaBH_4_/ScF_3_ composite is difficult from the thermodynamic point of view.^52^

During the rehydrogenation process, the experimental results show that only partial dehydrogenated products (NaBF_4_ + ScB_2_ + Na_3_ScF_6_) have reversibility, while the final dehydriding products (NaF + ScB_2_ + B) cannot be rehydrogenated. Apart from the thermodynamic factors, this might also be understood from structural similarity between [BF_4_]^−^ in NaBF_4_ and [BH_4_]^−^ in NaBH_4_, which may facilitate the regeneration of NaBH_4_ through the exchange between H^−^ and F^−^. The structural similarity was also observed between dehydrogenated and rehydrogenated products in the 3NaBH_4_/LnF_3_ systems, which led to the improved rehydrogenation kinetics in these systems.^25^ Researchers have also found that substitution reaction could be well understood from the hydride–fluoride isostructure, which has been proposed and confirmed in various hydrides–fluorides compounds having different stoichiometries.^59–61^

## 4 Conclusions

In this study, the 3NaBH_4_/ScF_3_ composite was prepared through mechanical milling. The behaviors and mechanisms of hydrogen de/absorption of the composite were explained by using TPD, DSC/TG, XRD and FTIR techniques. Following are the summarized results:

(1) TPD and DSC analyses confirmed that NaBH_*x*_F_4−*x*_ compound formed at the early dehydriding stage due to the partial substitution of H^−^ anion by F^−^ anion in NaBH_4_, releasing about 0.19 wt% of hydrogen. When temperature further increases, Na_3_ScF_6_, NaBF_4_ and ScB_2_ formed through the reaction between NaBH_4_ and ScF_3_ with 2.52 wt% of hydrogen released. Finally, the reaction of residual NaBH_4_ with Na_3_ScF_6_ produces NaF, B and ScB_2_, releasing about 2.83 wt% of hydrogen.

(2) The partially dehydrogenated products, Na_3_ScF_6_, ScB_2_ and NaBF_4_, can be rehydrogenated to generate NaBH_4_ with an activation energy of 44.58 kJ mol^−1^ H_2_. In contrast, the fully dehydrogenated products, NaF + ScB_2_ + B, cannot be hydrogenated. The hydrogen sorption reversibility of the partially dehydrogenated composite can be understood through thermodynamic point of view and the structural similarity between [BF_4_]^−^ in NaBF_4_ and [BH_4_]^−^ in NaBH_4_.

## Conflicts of interest

There are no conflicts to declare.

## Supplementary Material
